# Effect of Foaming Formulation and Operating Pressure on Thermoregulating Polyurethane Foams †

**DOI:** 10.3390/polym13142328

**Published:** 2021-07-15

**Authors:** Angel Serrano, Ana M. Borreguero, Juan Catalá, Juan F. Rodríguez, Manuel Carmona

**Affiliations:** 1 Department of Chemical Engineering, Institute of Chemical and Enviromental Technologies, University of Castilla-La Mancha, Av. Camilo José Cela s/n, 13004 Ciudad Real, Spain; aserrano@cicenergigune.com (A.S.); anamaria.borreguero@uclm.es (A.M.B.); juan.catala@uclm.es (J.C.); juan.rromero@uclm.es (J.F.R.); 2 Centro de Investigación Cooperativa de Energías Alternativas (CIC energiGUNE), Basque Research and Technology Alliance (BRTA), Parque Tecnológico de Alava, Albert Einstein 48, 01510 Vitoria-Gasteiz, Spain

**Keywords:** rigid polyurethane foam, phase change material, microcapsules, latent heat, energy storage, thermoregulation

## Abstract

The synthesis of rigid polyurethane (RPU) foams containing thermoregulatory microcapsules has been carried out under reduced pressure conditions with a new foaming formulation to reduce the final composite densities. These optimized RPU foams were able to overpass the drawbacks exhibited by the previous composites over the studied temperature range, working as insulating and thermal energy storage materials. The change in the formulation allowed to decrease the final foam density and enhance their mechanical strength. The effect of the operating pressure (atmospheric, 800 mbar, and 700 mbar) and microcapsules content (up to 30 wt%) on the physical, mechanical, and thermal PU foam properties was studied. The reduction of the pressure from atmospheric to 800 mbar did not have any effect on the cell size, strut thickness, and compression strength 10% of deformation, the Young modulus being even higher at 800 mbar. Nevertheless, a strong impact on the microstructure and mechanical properties was observed for the foam composites obtained at 700 mbar. A deleterious impact on the RPU foams thermal conductivity was observed when using low-pressure conditions. Thermal analyses showed that a composite able to work as heat accumulator and thermal insulation both at transient and at steady state was achieved.

## 1. Introduction

In the European Union, 64% of total energy consumption in the residential sector is used for heating and 15% for air conditioning. Therefore, this sector is a vital segment to carry out the energy transition currently demanded by leading global political and socio-economic actors [1,2]. Nevertheless, the presence of renewables as a source of energy in this sector is still below the necessary levels, so it is vital to promote its electrification. The direct use of renewable energies such as solar thermal and increased energy efficiency would be ideal for reaching where electrification cannot.

To achieve this higher energy efficiency and increase the penetration of solar thermal energy, innovative technologies must be applied and integrated into the “sustainable building” concept [3]. One of the most relevant of these emerging technologies is the use of phase change materials (PCMs) for storing and releasing large amounts of energy by means of latent heat when the temperature undergoes or overpasses its melting point [4,5,6]. Concerning their application in buildings, thermal storage systems based on PCMs can be classified as passive or active systems. In passive systems, PCMs are usually integrated into the building elements themselves, such as gypsum panels, bricks, polyurethane foams, concrete, or cement [7,8,9,10,11,12,13,14,15,16,17,18]. These elements primarily have a thermoregulatory function, preventing rooms from overheating during the day in the warm months or reducing the need for heating at night in the winter.

Among these building elements, rigid polyurethane (RPU) foams present the advantage of being a good insulating material due to its low thermal conductivity (the lowest of insulation materials along with polyisocyanurate). For that reason, in recent years many efforts have been focused on obtaining a material by combining microencapsulated PCMs with rigid polyurethane foams [15,16,19,20,21,22].

RPU foams synthesis is mainly based on the condensation reaction between diols or polyols and diisocyanates or polyisocianates in the presence of a catalyst. This reactive process can be simplified considering the two main chemical reactions that take place in the foaming process: isocyanate reactions with polyol hydroxyl groups—crosslinking reaction—and with water to produce CO2—blowing reaction [23,24]. Catalysts used to control the foaming process can promote the above reactions in different rates. Whereas organometallic catalysts promote crosslinking reactions, enhancing the foam structure, tertiary amines can catalyze both processes, and mainly the blowing reaction [25,26].

In a previous work, RPU foams containing up to 50 wt% of microcapsules containing a paraffin wax as PCM -mSD-(LDPE·EVA-RT27)- were developed [19,20]. These foams were synthesized using Tegoamin 33 as catalyst, which promotes both blowing and crosslinking reactions, and with a fixed quantity of surfactant and isocyanate. The results showed that, for mSD-(LDPE·EVA-RT27) contents greater than 30 wt%, foams are not able to retain enough amount of the inside generated CO2, increasing the density and thermal conductivity of the final composite. These changes produced a composite with a large TES capacity but worse properties as insulating material in the steady state, which is undesirable for energy savings. Moreover, the measurements carried out at full-scale revealed the detriment of the insulating capacity of this kind of composites compared with a standard RPU foam, which is also an important thermal property to enhance the energy savings in buildings. Hence, the ideal RPU foam-PCM composite must present high TES capacity which preserves a low thermal conductivity.

Previous studies determined that the use of Tegoamin BDE as catalyst allows obtaining PU foams with lower densities than those synthesized with Tegoamin 33, since Tegoamin BDE promotes only the blowing reaction and, thus, high foam heights are achieved. The reduction of foam density could reduce the thermal conductivity. Unfortunately, it was also observed a spoilage of the mechanical properties when Tegoamin BDE was used [24]. With the aim of decreasing the density of RPU foams containing microcapsules while keeping a proper mechanical resistance, the use of a mixture of both catalysts, Tegoamin 33 and Tegoamin BDE, should be explored.

On the other hand, a special foaming technique called Variable Pressure Foaming (VPF) can be used in order to keep or even decrease the thermal conductivity of the RPU foams. In VPF, the foaming process is performed under controlled low-pressure conditions, allowing to regulate the density and thus, custom-made foams can be produced [27]. This method is already industrially applied by companies such as FXI Innovations (USA) [28] or Interplasp (Spain) [29]. Apart from the detailed control of the final properties of the product, the use of VPF involves some additional advantages as the possibility of disesteem the use of blowing agents as well as the total capture of the residual gas emissions. As far as we know, the use of this technique for producing RPU foams-PCM composites having the above commented thermal purposes have not been published. Besides, there is a lack of information related with the effect of pressure on the properties of RPU foams synthesized at those conditions. Previous studies showed that, under vacuum conditions, the mixing of the initial raw materials for the foaming can be impeded by the boiling temperature of the liquid components, producing fragile foams [30].

Therefore, in the present work, the incorporation up to 30 wt% of mSD-(LDPE·EVA-RT27) to RPU foams using a mixture of Tegoamin 33 and Tegoamin BDE has been carried out at three different pressures (atmospheric, 800 and 700 mbar). The effect of the pressure and the microcapsules content on the physical and mechanical properties of the synthesized thermoregulatory RPU foams was analyzed. The thermal characterization of the specimens was also carried out to demonstrate the positive synergy for saving energy that can be achieved by using composites having low thermal conductivity and high TES capacity [31].

## 2. Materials and Methods

### 2.1. Materials

Polyol used in this work was R-4520 from Repsol YPF S.A. (Puertollano, Spain). Polymeric methylene diphenyl diisocyanate (PMDI) was supplied by AISMAR S.A. (Ribaforada, Spain). The catalysts used were Tegoamin 33 and Tegoamin BDE and the surfactant was Tegostab B8404, all supplied by Evonik Degussa International AG (Barcelona, Spain). Deionized water was used as blowing agent. Spherical microcapsules containing Rubitherm^®^RT27 with a shell from LDPE and EVA-mSD-(LDPE·EVA-RT27)- were obtained following the method described in the Patent EP2119498 [32] in a spray drying pilot plant. They have an average particle size of 10 μm and a latent heat of 98.14 J/g.

### 2.2. RPU Foams Formulation

**Figure 1:**
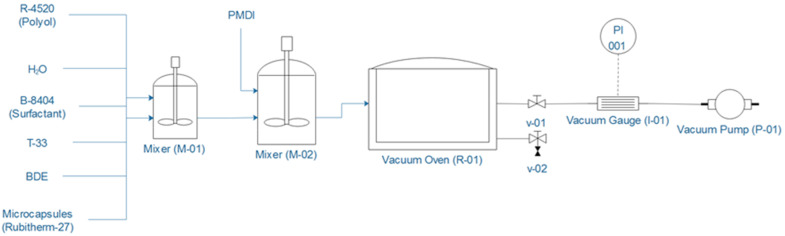
RPU foams synthesis facility scheme.

RPU foams were synthetized in a prismatic mold with dimensions of 20 × 20 × 13 cm^3^, by first weighting the desired masses of the polyol, silicone, water, catalysts, and mSD-(LDPE·EVA-RT27), and further stirring the mixture during 1 min at 1000 rpm. Then, the corresponding mass of isocyanate was added to the mixture and the resulting solution was stirred for just 5 s at 2000 rpm, at which moment the foam started to grow up. To control the pressure during the growth period, foams are introduced in a Nüve EV 018 vacuum oven (Henderson Biomedical, London, UK), fitted with a vacuum pump and a vacuometer DIVATRONIC DT1 (Leybold GmbH, Köln, Germany) for pressure regulation. Figure 1 shows a scheme of the facility for the RPU foams synthesis.

In a previous work composite foams containing between 0 to 50% by weight of mSD-(LDPE·EVA-RT27) were synthesized, achieving a TES capacity of 3.06 kWh/m^3^ with a latent heat of 34.4 J/g [19]. However, these foams presented an overly high density (>100 kg/m^3^) as well as high thermal conductivity when contents higher than 20 wt% of microcapsules were used (>0.07 W/m°C), important handicaps for considering their application. In order to overcome the weaknesses of these materials, a mixture of Tegoamin 33 and Tegoamin BDE was used as catalyst in this work; the amount of surfactant was varied with the content of added microcapsules, and three different pressures (atmospheric, 800 and 700 mbar) were studied. Moreover, the amount of isocyanate was recalculated according to the functionality of the polyol R-4520, by means of the Equation (1) [33].

(1) $$g_{PMDI}=\left[0.24*\left(IOH+Acidity\right)+15\cdot \left(g_{H_{2}O}+\%Humidity\right)\right]\cdot \frac{Ii}{100}$$

where *g_PMDI_* are the grams of PMDI per 100 g of polyol; *IOH* the hydroxyl number in mg KOH/g of the polyol; *Acidity* the acidity in mg KOH/g of the polyol; g_H2O_ are the grams of added water as reagent per 100 g of polyol; *%Humidity* is the water content of the polyol and *Ii* the isocyanate index.

The isocyanate index is defined according to the Equation (2).

(2) $$Ii=\frac{Actual\;amount\;of\;Isocyanate}{Theoretical\;amount\;of\;Isocyanate}*100$$

The amount of PMDI was set at an isocyanate index of 107, since isocyanate index values from 105 to 125 ensure the complete reaction of the hydroxyl groups and at the same time prevent the formation of allophanates, which appear with large excesses of NCO groups in the reaction mixture [34].

Table 1 summarizes the formulation used for the synthesis of a foam without microcapsules.

The quantity of the microcapsules added for each different percentage is calculated by means of Equation (3).

(3) $$g_{PCM}=\frac{g_{reference\;}\cdot \;\%PCM}{100-\%PCM}$$

where *g_reference_* is the amount of the rest of the compounds for the case of foams without microcapsules.

The amount of silicone, the surfactant chosen in the formulation, was found to be function of the quantity of added microcapsules with a 0.006; 0.0185; 0.024; 0.025 and 0.026 mass ratio respect to the polyol for the foams containing 0, 10, 20, 25, and 30 wt% of microcapsules, respectively. These silicon contents ensure the homogeneous dispersion of the particles into the foam.

The nomenclature designated for the composites was FoamPressure-mSD-(LDPE·EVA-RT27)%, where the subscript Pressure is the operational pressure used for the foam synthesis and *mSD-(LDPE·EVA-RT27)%* the percentage of embedded microcapsules. This way, a composite synthetized at 800 mbar and with 20 wt% of microcapsules would be named as Foam800-20%.

### 2.3. RPU Foams Characterization

#### 2.3.1. Apparent Density

Three cubic samples from the zones up, middle, and down of each synthesized foam with dimensions of 3 × 3 × 3 cm^3^ were taken out and weighted to calculate the average density (ρ) in kg/m^3^.

#### 2.3.2. Scanning Electron Microscopy Analysis

Synthesized RPU foams were depicted by scanning electron microscopy (SEM) to visualize possible changes in their cellular structure and also the mSD-(LDPE·EVA-RT27) location when different amounts of microcapsules are added. The average cellular size and the strut thickness were determined by the software Motic Image Plus 2.0. Three samples from each microcapsules content were analyzed with at least 25 cells per sample. To carry out these studies, a scanning electron microscope, model FEI QUANTA 200, with tungsten filament and a working potential between 5–30 kV was used.

#### 2.3.3. Mechanical Test

Compressive properties of foams were analyzed according to standard ASTM D1621 for rigid cellular plastics. Uniaxial compression tests were performed using an MTS 370.02 testing instrument. The compression tests were carried out at a cross- head speed of 2.5 mm/min. The tested foam specimens were prism of 5.1 × 5.1 × 2.6 cm^3^ and each analysis was repeated 3 times. From these test results their compressive stress at 10% deformation and the Young’s modulus (E*) were determined, this last one as the slope value of the initial part of the compression curves [35].

#### 2.3.4. Microcapsules Distribution and Average Latent Heat of Foams

The homogeneity of microcapsules distribution into the foam was studied in micro-scale by differential scanning calorimeter (DSC) analysis of samples taken out from three different zones: up, middle, and down. Three samples from each zone were analyzed. They were cubic shaped and with a weight close to 6.0 mg. The microcapsules presence and its amount into the foam can be obtained by the area under the peak observed at the PCM melting point. DSC analyses were performed in the range from −15 °C to 40 °C at a heating rate of 5 °C/min.

#### 2.3.5. Thermal Behavior

The thermal behavior of foams with microcapsules has been studied using the homemade equipment described and proved in previous works [36,37].

**Figure 2:**
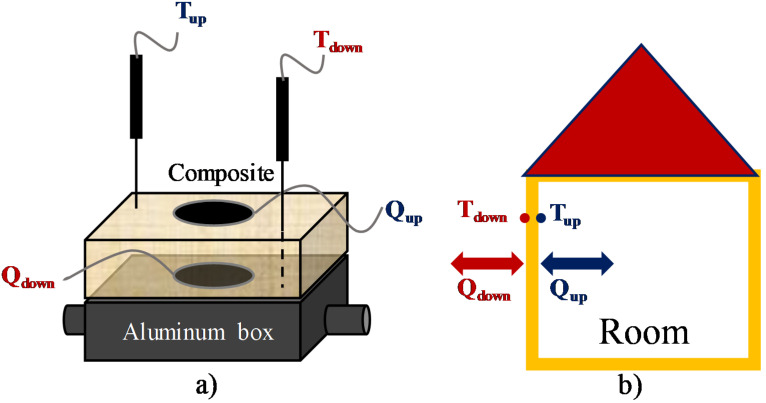
Scheme of the thermal behavior test (**a**), and its correlation with a dwelling (**b**).

Tests were carried out applying a thermostatic bath set-point step change from 18 to 40 ± 0.1 °C while temperatures at different foam sample locations and the inlet and outlet heat fluxes were registered with time. The foams samples dimensions were of 3 × 6 × 10 cm^3^. Four thermocouples of K-type were in the foam sample: two in the external sample surface (*T_up_*), and the other two on the aluminum cell (*T_down_*). The heat fluxes were measured by using gSKIN^®^-XI and gSKIN^®^-XP heat flow sensors for monitoring in real time the heat fluxes through each external face of the specimen. The temperature of the thermostatic fluid inside the insulated aluminum cell without foam sample is considered as the outdoor temperature. *T_down_* and *Q_down_* correspond to the temperature and heat flux that an outer surface of a house would register, whilst *T_up_* and *Q_up_* represent the surface temperature and heat flux in the indoor wall (Figure 2).

Using these signals, it is possible to quantify the TES capacity per cubic meter in a macro scale and the effective thermal conductivity at the final steady state (*k*) by means of Equations (4) and (5), respectively.

(4) $$\bar{TES}=\frac{q_{acc}}{m_{sample}}\cdot \frac{\rho }{3.6\cdot 10^{6}}\;$$

(5) $$k=\frac{Q_{in}\cdot X_{f}}{T_{down}-T_{up}}$$

where *q_acc_* is the amount of heat accumulated in the sample during the experiment (J) and it is calculated by subtracting the outlet to the inlet heat flux, *m_sample_* is the foam sample mass (kg), *ρ* is the foam density in kg/m^3^, $\bar{TES}$ is expressed in kWh/m^3^, *Q_in_* is the inlet heat flux at the final steady state condition (W/m^2^), and *X_f_* (m) is the foam thickness.

## 3. Results and Discussion

This section may be divided by subheadings. It should provide a concise and precise description of the experimental results, their interpretation, as well as the experimental conclusions that can be drawn.

### 3.1. Density

**Figure 3:**
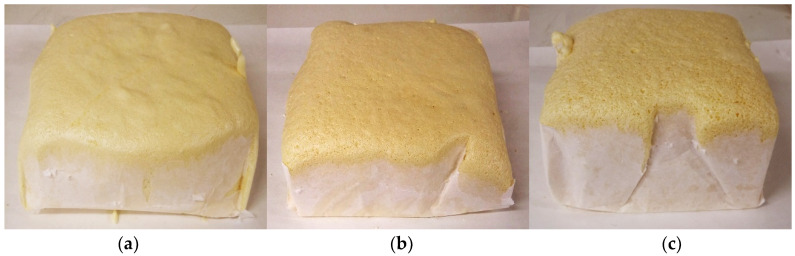
Photograph of the RPU foams containing a 20 wt% of microcapsules synthesized at different operating pressures: (**a**) atmospheric; (**b**) 800 mbar, and (**c**) 700 mbar.

As commented in the experimental section, three different pressures (atmospheric, 800 and 700 mbar) were studied, with the aim of reducing the foam density and consequently the thermal conductivity. Figure 3 shows the photographs of the RPU foams containing a 20 wt% of mSD-(LDPE·EVA-RT27) foamed at different operating pressures.

**Figure 4:**
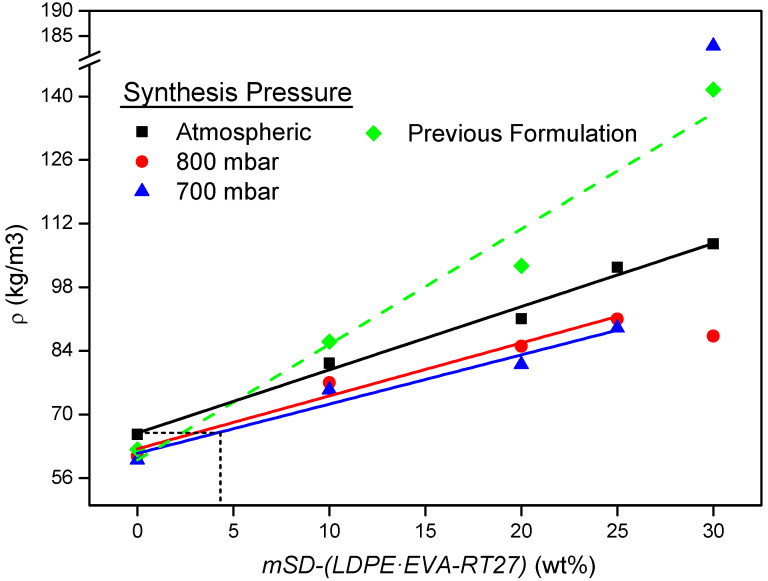
Apparent densities of the composites.

The apparent densities of RPU foams containing microcapsules and synthesized at different operating pressures were measured and represented in Figure 4. The results are compared with the density of the composites obtained with the previous formulation in which just catalyst Tegoamin 33 was used reported, in the work from Serrano et al. (2016) [19].

**Figure 5:**
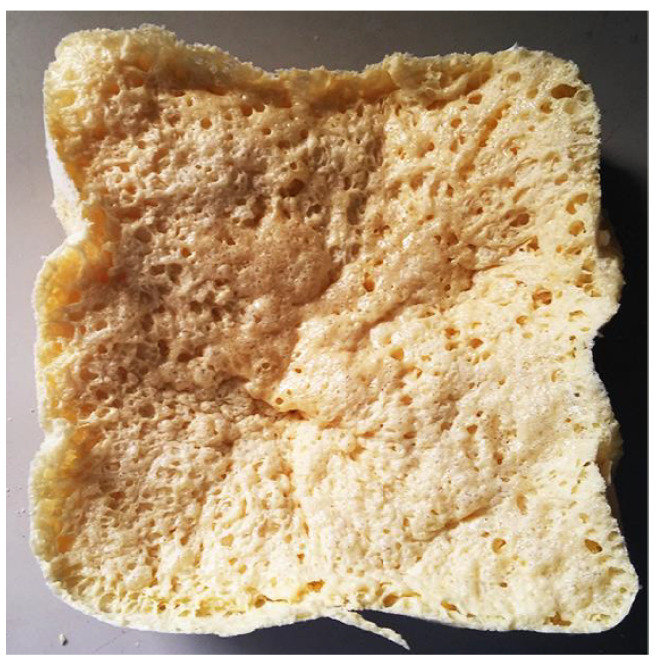
Appearance of Foam_700-30%_ after its collapse.

From Figure 4 it is evident that the new formulation allows to obtain lower densities for the same content of microcapsules, increasing this effect with the increase of the microcapsules addition. Foam_atm-30%_ exhibits a density 24% lower than that presented by its counterpart synthetized in the previous work. The decrease in the density is related with the use of Tegoamin BDE as catalyst, which promotes the blowing reactions, increasing the final height of the composite. Among the composites obtained with the optimized recipe, the higher the content of microcapsules and the operating pressure, the higher the apparent density of the foams. This response agrees with the results reported in literature using semi-vacuum conditions, where the low-pressure involves a higher foam expansion [27,30,38]. Although the density drop is pronounced when the pressure decreases from atmospheric pressure to 800 mbar, an additional reduction of 100 mbar up to 700 mbar does not involve a noticeable change in the density. In general terms, the increase of the density with the microcapsules content followed a linear trend, except for contents of 30 wt%. This content seems to be the addition limit when the operating pressure is decreased since, from this value, strong negative changes in the foaming process were observed. In case of the operating pressure of 700mbar, there was a sharply density increase due to the foam collapse (see Figure 5). For Foam_800-30%_, its density exhibited an unexpected collapse that could be ascribed to microstructural changes that do not allow the proper consolidation of the structure.

Hence, the decrease in the density of RPU foams must be related with changes in their microstructure, promoted by the operating pressure. According to Figure 4, it is also possible to affirm that RPU foams containing up to 4 wt% of microcapsules can be developed, having a similar density to that exhibited by foams without thermoregulatory materials and synthetized at atmospheric pressure. However, a content of 4 wt% of microcapsules is not high enough to ensure a large TES capacity of this kind of material.

### 3.2. Foam Microstructure Characteristics

**Figure 6:**
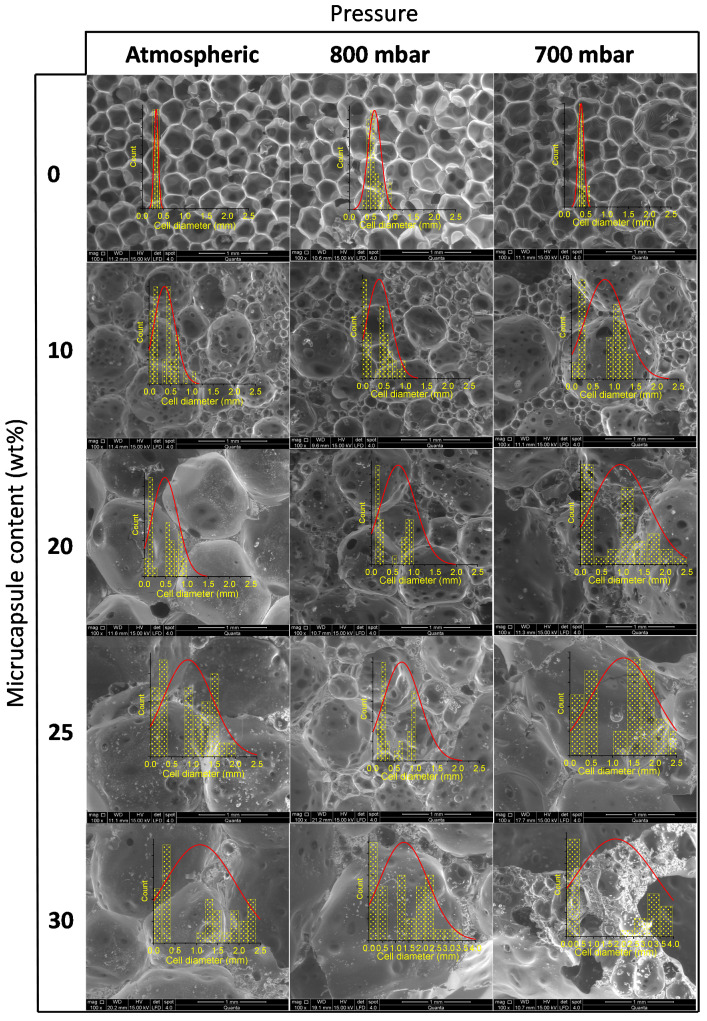
SEM photos of the internal foams synthesized under different pressures and contents of microcapsules.

**Figure 7:**
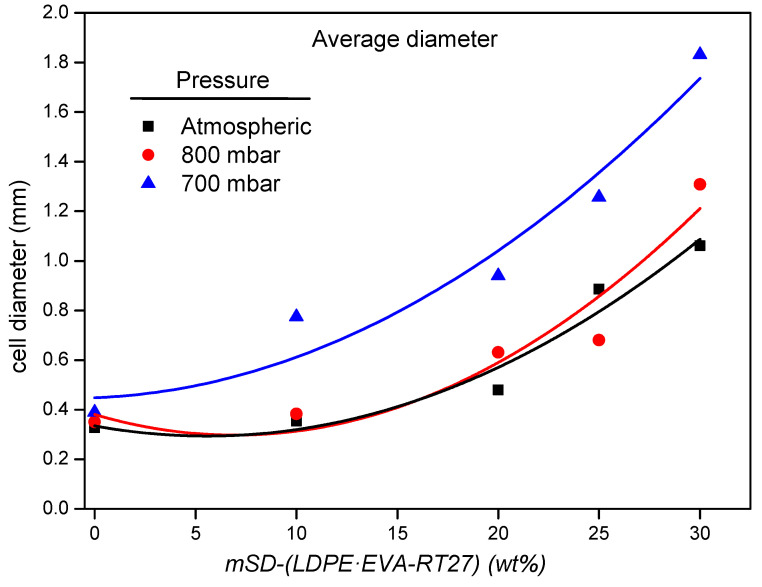
Average cell diameter of the obtained foams for the different operational pressures.

**Figure 8:**
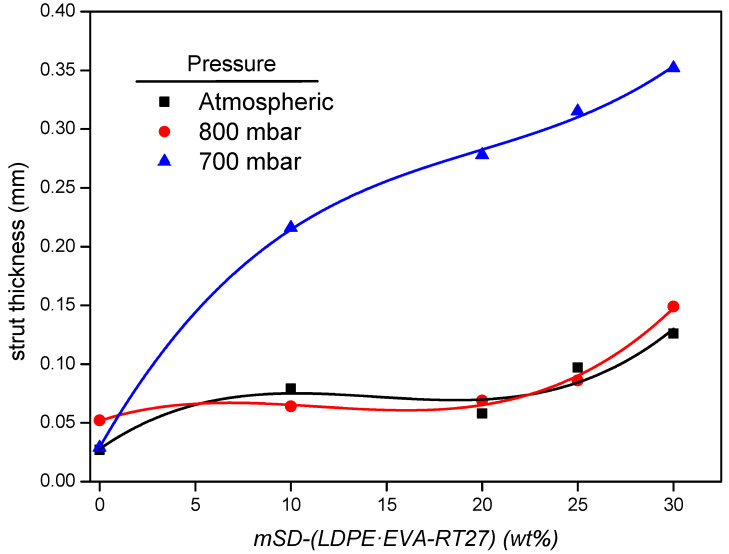
Strut thickness of the obtained foams for the different operational pressures.

Understanding how the operating pressure influences the microstructure of the composite is crucial to explain the changes in the foam’s density. SEM photographs of the foam cells are shown in Figure 6 together with their cell size distribution, measured from each image by using the software Motic Image Plus [39]. As can be seen, foams without microcapsules presented a homogenous distribution of cell diameters. For these foams, some folds appear on the wall cells as the operating pressure decreases, this effect being more noticeable for the foam synthesized at 700 mbar. This anomalous wall appearance could be as consequence of the cell struts distension, caused by the depression peaks arisen as result of the higher vacuum conditions. As the microcapsule content increases, the cell size distribution becomes wider due to the appearance of a second family of larger cells. Figure 7 and Figure 8 show the average cell size and the strut thickness for the synthesized RPU foams, respectively.

As can be observed in Figure 7, the cell size of Foams_700_ is significantly larger for the same microcapsules content than that obtained with higher operating pressures, which agrees with the above-mentioned greater expansion of the foam at reduced pressure conditions. This large cell size of the RPU foams synthesized at 700 mbar could promote a decrease in the mechanical properties. On the other hand, the cell sizes of Foams_atm_ and Foams_800_ follow a similar trend with the microcapsules addition, thus, similar mechanical properties can be expected.

Besides, the mechanical strength of the foams is strongly dependent on the strut characteristics. As can be seen in Figure 8, the strut thickness of Foams_700_ increased sharply with the microcapsules addition, being considerably higher than the displayed for Foams_800_ and Foams_atm_, which have similar strut thicknesses in all the range of PCM incorporation. The greater strut thickness of Foam_700_ is mainly due to the existence of enclosed gas bubbles inside the strut instead of solid polyurethane polymer (Figure 6), probably due to the raw materials vapor bubble formation at that low-pressure condition. The presence of enclosed gas bubbles is in agreement with the commented weakness of the foam struts when 700 mbar of pressure is used for the composite synthesis. Hence, these microstructure analyses could predict a sharp deterioration in the mechanical properties of the composites synthetized at 700 mbar, and a lower effect on the foams obtained at 800 mbar.

### 3.3. Mechanical Behavior of the Composite Foams

**Figure 9:**
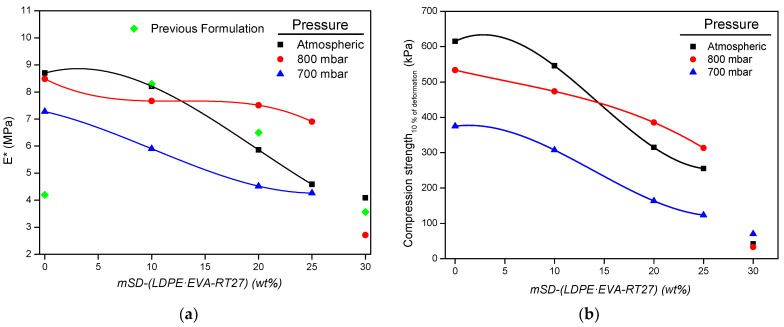
Young´s modulus (**a**) and compression tension at 10% deformation (**b**) for the composite foams synthetized at different operating pressures.

To verify the proper mechanical behavior of the samples, Young’s modulus of the composite foams (E*) and their compressive stress at 10% deformation were determined. It is well known the foam mechanical strength dependency on its density and cell size, decreasing for larger cell sizes and lower densities [23,40,41]. The obtained values are shown in Figure 9, where the Young’s modulus obtained with the previous formulation is also depicted.

It should be initially noted from Figure 9a that, in the RPU foams without microcapsules, the obtained E* values were greater than that of previous formulation, which means that the use of both Tegoamin 33 and Tegoamin BDE catalysts and the adjustment of surfactant and polyol:isocyanate ratio made in the foam formulation enhanced the mechanical strength of the obtained composites. On the other hand, E* and compressive stress at 10% deformation decrease both with the number of embedded microcapsules and when using 700 mbar of operating pressure. The reduction of the mechanical strength due to reduced pressure operating conditions is directly related to the large cell sizes, the occurrence of folds on the foam cell walls, and the presence of gas bubbles mainly in Foam_700_ struts. Likewise, the detriment of Young´s modulus of foams with the microcapsules content is consistent with results reported in literature [24], and it is also related with the growth of the foam cell size with the particles content. Despite mechanical properties in Foam_atm_ decreasing abruptly for microcapsules content above 10 wt%, Young´s modulus in Foam_800_ remains practically equal up to 25 wt% of microcapsules. Finally, there was a dramatic drop in the mechanical strength for microcapsules content of 30 wt% associated with the sudden rise of the foam cell size that led us to discard them for further studies.

In accordance with the Spanish standard UNE-EN 14315-1 [42], which determines the specification for the thermal insulating products (RPU foams) for building applications, Foam_800-25%_ exhibits a compressive stress level of CS(10\Y)300. It means a compressive stress at 10% deformation higher than 300 kPa, which is higher than those of Foam_atm-25%_ and Foam_700-25%_, CS(10\Y)200 and CS(10\Y)100, respectively.

### 3.4. Microcapsules Distribution and Average Latent Heat of the Foams

**Figure 10:**
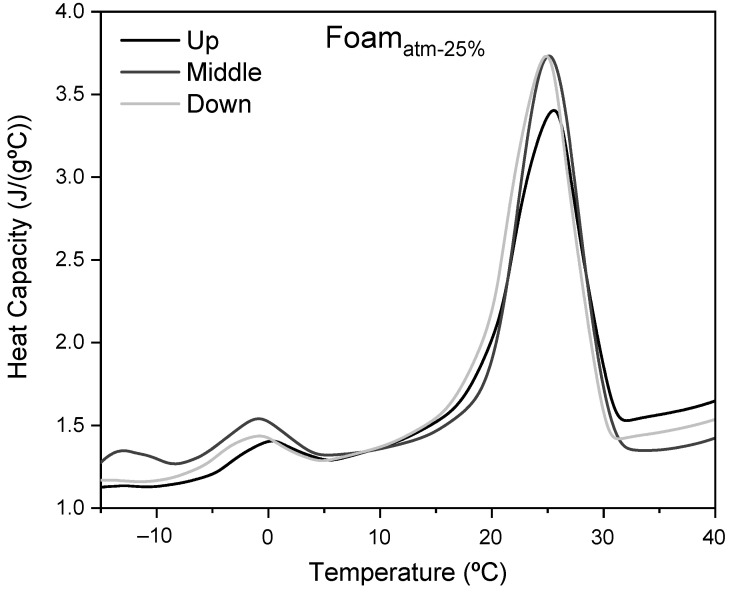
Heat Capacity of Foam_800-25%_ as function of the temperature at different heights.

The thermal behavior of this kind of foams, especially with high content of microcapsules, is affected by the dispersion of the particles inside the composite [43]. In order to study the microcapsules distribution, as well as their actual content in the foams, DSC analyses of samples taken out from three different zones were performed. As an example, Figure 10 presents the heat capacity of Foam_800-25%_ as a function of the temperature obtained by DSC from three different zones of the foam.

As can be seen, the thermograms obtained at different heights are similar to each other. Focusing on the main peak, the onset temperatures of the composite are in the range of 18.5 °C to 19.5 °C, with a temperature of maximum heat capacity from 24.4 °C to 25.7 °C. The latent heat of the samples can be obtained from the integration of the heat capacity profile. Based on the observations in all samples, no trend in the latent heat distribution along the foam has been found. Table 2 shows the average latent heat depending on the operating pressures and their standard deviations.

The small variation of the latent heat values for the different foam specimens demonstrated a homogeneous distribution of the microcapsules along the foam, with a maximum latent heat deviation of 16% (Foam_atm-10%_). On the other hand, as expected, a linear relationship was observed between the latent heat and the theoretical microcapsules content. The latent heat exhibited by the composite Foam_800-25%_ (22.6 J/g) was the highest one.

Thus, among the synthesized composites and bearing in mind the mechanical weakness of Foams_700_, the composites Foam_atm-25%_ and Foam_800-25%_ could be chosen as the better materials to be used in buildings attending to their high latent heat, proper mechanical strength, and low density.

### 3.5. Thermal Behavior

A fundamental issue to be analyzed is the thermal behavior of the selected foams as insulating and thermal energy storage material for being considered as an optimal element for saving energy in a final building application. For that purpose, the thermal test described in the experimental section was carried out.

**Figure 11:**
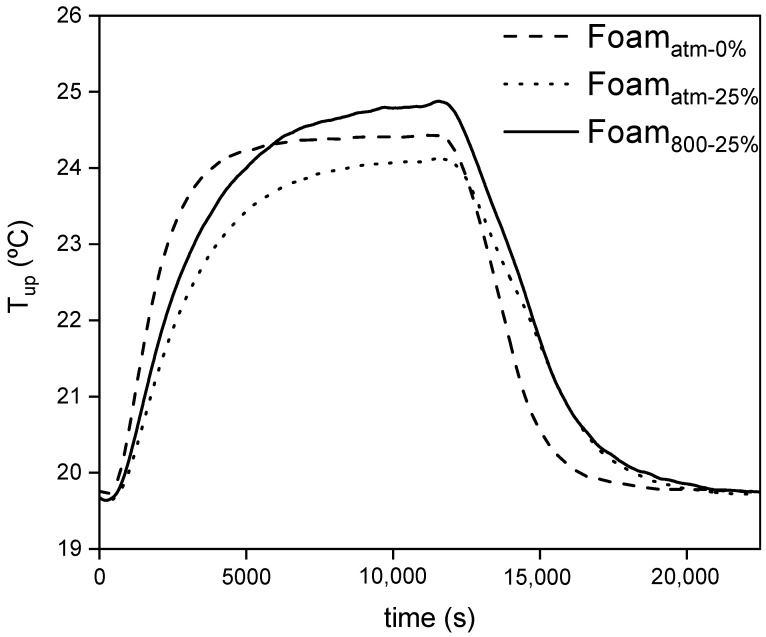
Temperature profiles of the external surface (*T_up_*) of the selected composites subjected to a heating-cooling cycle.

Figure 11 shows the *T_up_* of the selected composites (Foam_atm-25%_ and Foam_800-25%_) compared with the profile obtained for Foam_atm-0%_ as reference sample, when they were subjected to the heating process from 18 to 40 °C up to reach the steady state, and further cooling down to the new steady state at 18 °C. The steady states were reached in order to test the insulating properties of the foams.

As can be observed in Figure 11, at the transient state, Foam_atm-0%_ *T_up_* increased sharply during the heating process as well as decreased suddenly during the cooling step. However, the presence of microcapsules softened these temperature changes. Foam_atm-25%_ and Foam_800-25%_ exhibited a lower slope of the temperature profile at the transient state, causing a thermal damping effect which promotes a longer comfort time.

At the steady state of the heating process, *T_up_* of Foam_800-25%_ was the highest temperature of the studied composites. This behavior suggests an increase of the thermal conductivity of this material. On the contrary, Foam_atm-25%_ exhibited a lower *T_up_* than that of the control sample, indicating that its insulating capacity was enhanced. The loss of insulating capacity of Foam_800-25%_ despite its lower density could be related to an insufficient retention of the CO_2_ coming from the synthesis due to the low-pressure conditions [44,45]. The *T_up_* at steady state of the cooling cycle was similar for all the composites, which implies a preservation of the insulating capacity at low temperature.

**Figure 12:**
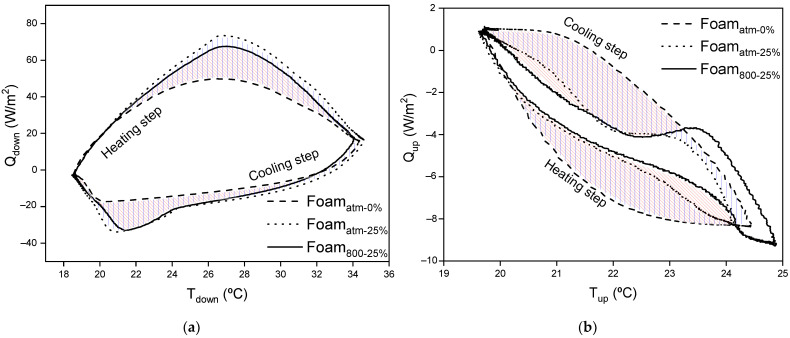
Variation of the input and output heat fluxes for a heating-cooling cycle as function of the outdoor or indoor temperature, (**a**) Q_down_ vs. T_down_ and (**b**) Q_up_ vs. T_up_.

The effect of the PCM on the foams is further evidenced in Figure 12, where the heat fluxes through the bottom (Q_down_) and the upper (Q_up_) surface of the samples respect to their corresponding *T_down_* and *T_up_*, respectively, are represented.

As can be observed in Figure 12a, the presence of microcapsules increased the dome shape of the incoming heat flux during the heating process, related to the larger energy storage capacity of the composites doped with microcapsules as previously commented. This is due to fact that the PCM is melting and storing the heat.

The peak temperature of maximum incoming heat flux during the heating step occurs at 26 °C for the composites containing microcapsules. During the cooling process the heat flux profile of Foam_atm-25%_ and Foam_800-25%_ presents an abrupt change at around 24 °C, as a result of the PCM charge process, which starts to solidify releasing the previously accumulated heat with a small change in its temperature. This process finishes at around 19.5 °C, when the temperature decreases below the onset temperature of the composite.

**Figure 13:**
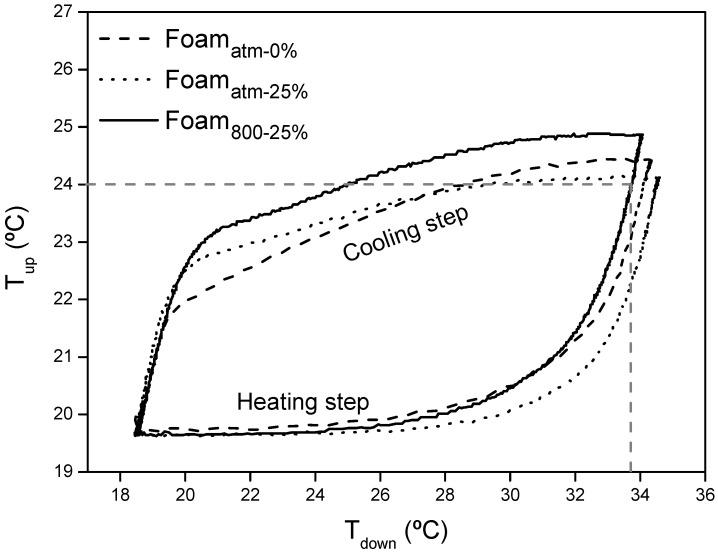
*T_up_* as function of *T_down_* registered in the thermal behavior test.

As aforementioned, Figure 12b represents the heat flux throughout the indoor surface of a wall, it means, the incoming or outgoing heat in a room. In that way, the negative heat flux values correspond to the heat transfer from the composite to the inside of a room. It is plain to see that, in the heating step at the transient state the highest incoming heat flux was exhibited by Foam_atm-0%_. On the contrary, the uptake of energy by the PCM in Foam_atm-25%_ (blue area in graph) and Foam_800-25%_ (red area in graph) softened the incoming heat until *T_up_* reached 24 °C. In this *T_up_*, the temperature registered on the bottom surface, *T_down_*, was 33.5 °C, as shown in Figure 13. Once that temperature was achieved, the greater portion of the composite had overpassed its offset temperature (Figure 10), having no capacity to uptake more energy and increasing in that way the incoming heat flux into the room. This effect predominated in Foam_800-25%_, the outcome of which is noticeable in Figure 11, where its temperature at steady state after the heating process was the highest one. Although the incorporation of microcapsules greatly reduced the incoming heat from the outside for Foam_atm-25%_ and Foam_800-25%_ during the transient states, at the steady state of the heating process only Foam_atm-25%_ preserved an improved insulation capacity.

During the cooling step, when *T_up_* was below 23.5–23.8 °C for Foam_atm-25%_ and Foam_800-25%_, *Q_up_* reaches a plateau region that finishes at 22.2 °C. This *T_up_* range covers a *T_down_* from ~28 °C to 19 °C in the cooling step, as depicted in Figure 13. Hence, the plateau area represents the solidification of the PCM, process in which the composite would transfer its previously stored heat to the inside of a room. In this way, the temperature profile of these composites dropped smoother than that exhibited by Foam_atm-0%_, without PCM (Figure 11), during the cooling step.

Based on these results, it can therefore be concluded that the new formulation and the incorporation up to 25 wt% of microcapsules not only improved the thermal damping effect but also allowed to improve the isolation capacity of the final composites. Besides, although the use of 800 mbar of operating pressure decreased the density of the obtained RPU foam, it had a deleterious effect on its insulating capacity, probably due to the loss of CO_2_ under low-pressure conditions.

To corroborate the previous conclusions, the effective thermal conductivity (*k*) of the above composites was estimated according to Equation (5) and the values are summarized in Table 3.

It is clear from the results that *k* of Foam_atm-0%_ and Foam_atm-25%_ remained low and equal between them. On the contrary, accordingly to the previous results, the thermal conductivity of Foam_800-25%_ has been increased, as is noticeable in the temperature profiles represented in Figure 11. Moreover, the *k* value for Foam_atm-25%_ was clearly lower than the one reported in the previous work [19] for 30 wt% of microcapsules content (0.073 W/m°C). These results corroborate the improvement achieved with the new foaming formulation, decreasing the final composite density, and enhancing their insulating capacity compared with the previous recipe. Nevertheless, the use of low-pressure conditions damages the thermal properties of the RPU foam composites containing microcapsules.

Additionally, from the thermal analyses, the thermal energy storage capacities per cubic meter (TES capacities) for the optimal composite and for the control sample were determined. The TES capacities of Foam_atm-0%_ and Foam_atm-25%_ were 0.57 and 1.43 kWh/m^3^, respectively. Thus, the TES capacity of the developed composite was 2.5 times higher than that of a conventional foam.

Hence, these results demonstrated that using a catalyst mixture that favors the blowing reaction and adjusting the silicon and isocyanate index allowed to obtain composite foams that fulfil a dual purpose, heat storage, and thermal insulation at transient and steady state.

## 4. Conclusions

The synthesis of a RPU foam which combines, over the whole studied temperature range, both thermal energy storage and insulating capacities by modifying the foaming formulation has been demonstrated.

The use of low-pressure operating conditions for the foam synthesis allows to produce a foam containing up to 4 wt% of microcapsules having a similar density than that exhibited by foams without thermoregulatory materials and synthetized at atmospheric pressure. A high increase in the cell size and strut were observed for the foam composites obtained at 700 mbar which was deleterious attending to mechanical purposes. Regarding the thermal analyses, although the use of low-pressure conditions promotes lower densities and proper mechanical strengths up to 800 mbar, it has a deleterious impact on the RPU foams thermal conductivity, probably due to the release of the CO_2_ formed during the foaming reaction.

It can be concluded that the use of the proper amount of surfactant, suitable isocyanate:polyol mass ratio, and a combination of Tegoamin BDE and Tegoamin 33 for the synthesis of RPU foams containing 25 wt% of microcapsules allowed to obtain a composite with a TES capacity of 1.43 kWh/m^3^ and an effective thermal conductivity of 0.048 W/m°C. These properties result in a composite with temperature damping capacities in the transient state while keeping a proper insulating capacity. Moreover, the mechanical strength showed by Foam_atm-25%_ makes this material suitable to be use as envelope in buildings for increasing their energy efficiency and reducing the contribution of the buildings to the CO_2_ emissions and global warming in general. Finally, it can be stated that the Variable Pressure Foaming is useful for the density variation of the RPU foams, but the adjustment of the foam formulation is more efficient for the improvement of their thermal and mechanical properties.

## Figures and Tables

**Table 1:** Mass ratio of the reactants for the synthesis of polyurethane foams without microcapsules.

Reagent	Mass Ratio
Polyol: R-4520	1
Surfactant: Silicone Tegostab 8404	0.006
Blowing Agent: H_2_O	0.0164
Catalyst 1: Tegoamin 33	0.0182
Catalyst 2: Tegoamin BDE	0.0164
Isocyanate: PMDI	1.455

**Table 2:** Latent heat from DSC analysis and standard deviations of the measurements.

Microcapsules (wt%)	Latent Heat (J/g)		
	P_atm_	800 mbar	700 mbar
10	6.0 ± 1.0	5.1 ± 0.5	4.5 ± 0.6
20	12.7 ± 0.3	12.2 ± 1.2	14.0 ± 1.7
25	20.3 ± 1.1	22.6 ± 2.6	18.7 ± 1.8

**Table 3:** Thermal conductivity of the synthetized composite foams.

Composite	*k* (W/m°C)
Foam_atm-0%_	0.048
Foam_atm-25%_	0.048
Foam_800-25%_	0.057
